# Supplementary material for: Accessory proteins of the RAS-MAPK pathway: moving from the side line to the front line
Source: Commun Biol. 2021 Jun 8;4:696. doi: 10.1038/s42003-021-02149-3 (PMC8187363; doi:10.1038/s42003-021-02149-3)
Supplement: Supplementary file 2 — Supplementary Information [file 42003_2021_2149_MOESM2_ESM.pdf]

Supplemental Table 1. Abbreviation list

AKT	Protein kinase B
ANK	ankyrin repeats
APC	Antigen-presenting cell
ASPP	apoptosis-stimulating of p53 protein
ARR N	arrestin N-terminal domain
ARR C	arrestin C-terminal domain
BCR	B cell antigen receptor
BCR-ABL	breakpoint cluster region-Abelson tyrosine kinase
C	cysteine-rich
CaM	Calmodulin
CaMII	Calmodulin kinase II
CAV1	Caveolin-1
CBL	casitas B-lineage lymphoma proto-oncogene
CC	coiled coil
CD3	T-cell surface glycoprotein CD3
CHD	calponin homology domain
CNK1	connector enhancer of kinase suppressor of RAS 1
CRD	carbohydrate-recognition domain
CRIC	conserved region in CNK
CRKL	CRK-like protein
CT	C-terminal domain
DE	aspartic acid and glutamic acid-rich
DED	death effector domain
DHR	DLG homologous region
DOK	docking protein
EF	EF-hand
EGF	epidermal growth factor
EGFR	EGF receptor
Erbin	Erbb2-interacting protein
ERK	extracellular signal regulated kinase
ERM	ezrin-radixin-moesin
EVH	Ena-VASP homology
FAK	focal adhesion kinase 1
FERM	4.1 protein/ezrin/radixin/moesin domain
FGF	fibroblast growth factor
FHL	four and a half LIM domains protein
FLOT	Flotillin
FRS2	fibroblast growth factor receptor substrate 2
GAB	GRB2-associated-binding protein
GADS	GRB2-related adapter protein 2
GAL	Galectin
GAP	GTPase-activating protein
GAR	GAS2-related domain
GDP/GTP	guanosine di/triphosphate
GEF	guanine nucleotide exchange factor
GF	growth factor
GPCR	G protein-coupled receptor
GPR	G-protein regulator
GRB2	growth factor receptor binding protein 2
GRD	GAP-related domain
GRF1	guanine nucleotide release factor 1
GRK2BD	GRK2 binding domain
HBD	Histone binding domain
IDRs	intrinsically disordered regions
IRS	Insulin receptor substrate
IQ	IQ motif
KBD	KIT-binding domain
KSR1	kinase suppressor of RAS-1
L	leucine-rich
LAT	linker for activation of T-cells family member 1
LCK	Lymphocyte cell-specific protein-tyrosine kinase
LD	leucine-rich sequence motif
LIM	LIN-11/Isl-1/MEC-3 domain
LPS	lipopolysaccharide
LLPS	liquid-liquid phase separation
LRR	leucine-rich-repeats
MAPK	mitogen activated protein kinase
MEK	Dual specificity mitogen-activated protein kinase kinase 1
MHC	major histocompatibility complex
MORG1	WD repeat domain-containing protein 83
MP1	MEK-binding partner 1
MTB	microtubule-binding region
NCL	Nucleolin
NES	nuclear export signal
NF1	neurofibromatosis type 1
NLS	nuclear localization signal

NoLS	nucleolar localization signal
NPM	Neucleophosmin
NSCLC	Non-small cell lung cancer
NTAL	non-T-cell activation linker
OD	oligomerization Domain
P	proline-rich
P14	14 kDa protein
P18	18 kDa protein
P85	regulatory subunit of phosphatidylinositol-3 kinase holoenzyme
PAK	p21-activated kinase 1
PAQR	Progestin and adipoQ receptor family member
PBS	paxillin binding site
PD	programmed death-1
PD-L1	programmed death-1 ligand
PDZ	PSD95/discs-large/zona occludens-1
PEA15	phosphoprotein enriched in astrocytes
PH	pleckstrin homology
PHB	prohibitin homologues
PIX	PAK interacting exchange factor
PI3K	phosphoinositide 3-kinase
PKC	protein kinase C
PM	plasma membrane
PMA	phorbol 12-myristate 13-acetate
PP1	protein phosphatase 1
PPP1CB	protein phosphatase 1 catalytic subunit beta
PTB	phosphotyrosine binding
PTP	phosphotyrosin phosphatase
Q	glutamine-rich
RAF	rapidly accelerated fibrosarcoma
RAS	rat sarcoma
RASGRP1	RAS Guanyl Releasing Protein 1
RBD	RAS binding domain
RGCT	RASGAP C-terminus
RGS	regulators of G protein signalling
RING	really Interesting New Gene finger domain
RKIP	RAF kinase inhibitor protein
RNA-BD	RNA binding domain
RRM	RNA recognition motif
RSK	ribosomal S6 kinases
RTK	receptor tyrosine kinase

S	serine-rich
SAM	sterile alpha motif
SEFIR	SEFs and IL17Rs domain
SH	SRC homology domain
SH2	SRC homology 2
SH3	SRC homology 3
SHC	SRC homology and collagen domain protein
SHD	SPA2 homology domain
SHP2	SH2 domain-containing tyrosine phosphatase 2
SID	SIN3 interaction domain
SOS	son of sevenless homolog protein
SPFH	Stomatin/ Prohibitin/ Flotillin/HflK/C
SPR	cysteine-rich sprouty domain
SPRED	Sprouty-related, EVH1 domain-containing protein
SPRY	Sprouty
TCR	T-cell receptor
TM	transmembrane domain
TRKA	tyrosine kinase receptor A
UBA	ubiquitin associated domain
UBL	ubiquitin-like domain
WDs	WD repeats
WH1	WASP Homology domain 1
WW	two tryptophan containing β-sheets
ZAP-70	zeta chain of T cell receptor associated protein kinase 70
4H	four-helix bundle
